# Precipitation strengthening in an ultralight magnesium alloy

**DOI:** 10.1038/s41467-019-08954-z

**Published:** 2019-03-01

**Authors:** Song Tang, Tongzheng Xin, Wanqiang Xu, David Miskovic, Gang Sha, Zakaria Quadir, Simon Ringer, Keita Nomoto, Nick Birbilis, Michael Ferry

**Affiliations:** 1 School of Materials Science and Engineering, The University of New South Wales, Sydney, NSW 2052 Australia; 2 Herbert Gleiter Institute of Nanoscience, School of Materials Science and Engineering, Nanjing University of Science and Technology, Nanjing, Jiangsu 210094 China; 3 Microscopy and Microanalysis Facility, John de Laeter Centre, Curtin University, Bentley, WA 6845 Australia; 4 School of Aerospace, Mechanical and Mechatronic Engineering and Australian Centre for Microscopy and Microanalysis, The University of Sydney, Sydney, NSW 2006 Australia; 5 Department of Materials Science and Engineering, Monash University, Melbourne, VIC 3800 Australia

## Abstract

Body-centred cubic magnesium-lithium-aluminium-base alloys are the lightest of all the structural alloys, with recently developed alloy compositions showing a unique multi-dimensional property profile. By hitherto unrecognised mechanisms, such alloys also exhibit exceptional immediate strengthening after solution treatment and water quenching, but strength eventually decreases during prolonged low temperature ageing. We show that such phenomena are due to the precipitation of semi-coherent D0_3_-Mg_3_Al nanoparticles during rapid cooling followed by gradual coarsening and subsequent loss of coherency. Physical explanation of these phenomena allowed the creation of an exceptionally low-density alloy that is also structurally stable by controlling the lattice mismatch and volume fraction of the Mg_3_Al nanoparticles. The outcome is one of highest specific-strength engineering alloys ever developed.

## Abstract (web summary)

Solution treatment and quenching can strengthen magnesium-lithium-aluminium alloys, but this strength decreases with ageing. Here, the authors show this is due to semi-coherent nanoparticle precipitation followed by coarsening, and control the lattice mismatch to stabilise the microstructure.

## Introduction

Magnesium (Mg) alloys are the lightest structural metallic materials (*ρ* ~1.4–1.9 g cm^−3^) and, as such, offer significant potential use in many technological, industrial and consumer marketplaces^1–7^. However, low strength and ductility, together with poor corrosion resistance and instability, have limited their wider applications. Ductility and corrosion resistance were improved considerably by our recent discovery of an ultra-low-density (1.4 g cm^−3^), body-centred cubic (BCC) magnesium-lithium-aluminium-base alloy after suitable processing^8^, and thereby low strength and instability remain to be the restrictive factors for the development of this class of alloys.

Mg-Li-Al alloys with composition ranges falling slightly outside our prototype alloy have been studied for over 60 years, but the precise role of Al on microstructural and mechanical behaviour has remained unascertain at the time^8–12^. For example, it was claimed originally that a metastable MgAlLi_2_ phase (L2_1_ ordered structure, space group: ${\mathrm{F}}\bar 43{\mathrm{m}}$, lattice parameter: ~6.7 Å) is the strengthening phase that subsequently transforms to the equilibrium AlLi phase on artificial ageing^13–15^. The former was first studied by X-ray diffraction (XRD) and assigned the tentative composition MgLi·LiAl^9^. This structure can be derived from the B32-AlLi crystal structure by replacing half of the Al atoms with Mg atoms. It was further proposed that either MgLiAl_2_ phase or some type of spinodal decomposition reaction were the cause of strengthening in Mg-Li-Al alloys with the subsequent precipitation of hexagonal close-packed (HCP) α-Mg causing age softening^10,16–18^. In such prior studies, XRD was the main technique for investigating the structural origins of strengthening and softening, and complementary quantitative compositional and crystallographic analysis methods had never been reported. Moreover, unlike any other age hardenable alloy system, such as Mg-Al, Al-Cu, Cu-Be etc.^1,19,20^, our Mg-Li-Al alloy appears to be unique in that a solution-treated condition reaches near peak strength either on water quenching or quickly thereafter. In contrast, as-quenched Al-free Mg-(10–15 wt.%) Li alloys do not show this immediate strengthening^21^.

Herein, we identify the nanoprecipitation behaviour and the associated mechanisms of immediate precipitation strengthening on quenching and subsequent age softening in Mg-Li-Al-base alloys and propose and validate a new methodology for generating more structurally stable alloys with specific strengths exceeding all but a handful of every major commercially available Mg-, Al-, Ti-, Co-, Cu- and Fe-based engineering alloy to date.

## Results

### Mechanical properties

**Fig. 1:**
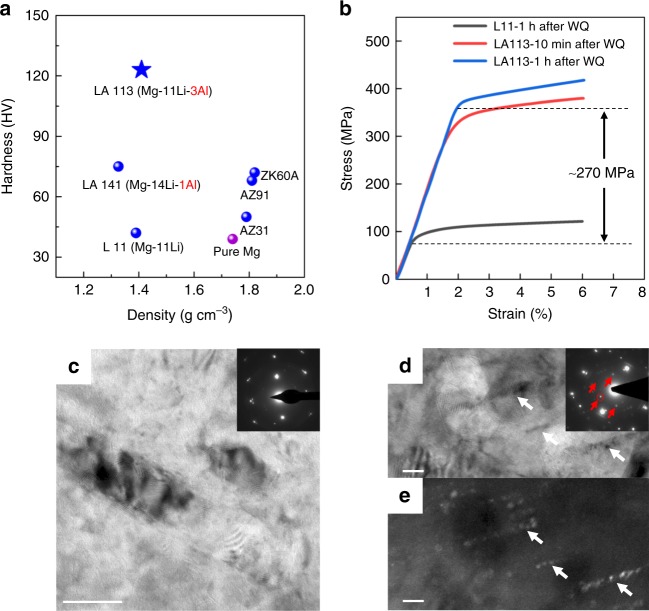
Mechanical properties and typical transmission electron microscope (TEM) images of L11 and LA113. **a** Vickers hardness as a function of density of selected water-quenched (WQ) Mg-Li-based alloys, compared with high purity Mg and some conventional commercial Mg alloys. **b** Ambient temperature stress–strain curves of L11 and LA113 tested in uniaxial compression after solution treatment at 400 °C and water quenching. **c**, **d** Bright-field TEM images of WQ L11 and LA113, respectively. The insets show the selected area electron diffraction (SAED) patterns of the corresponding microstructure indexed as <011>_β_. The red arrows show the superlattice diffraction spots corresponding to a body-centred cubic (BCC)-based D0_3_ ordered structure. **e** Dark-field image taken from the reflection marked by the red circle in **d**. Typical rod-like precipitates are marked by white arrows in **c**, **d**. Scale bar, 50 nm in **c** and 20 nm in **d**, **e**

Figure 1a shows the hardness/density section of property space of selected Mg-Li-based alloys in the solution-treated and water-quenched (WQ) state, compared with high purity magnesium and some typical commercial Mg alloys. For the Mg-Li-Al alloys, increasing the Al content increases hardness significantly, which is consistent with previous reports^10^. Hence, modest additions of Al to binary Mg-Li alloys generates a hardness exceeding many standard commercial Mg alloys without significantly affecting the ultra-low density of the former. Figure 1b shows representative flow curves in uniaxial compression for a (wt.%) Mg-11Li binary alloy (designated as L11) and Mg-11Li-3Al-1(Zr, Y) alloy (designated as LA113) after solution treatment for 10 min at 400 °C, followed by water quenching (see Methods). The compressive yield (0.2% proof) stress for LA11 and LA113 within 60 min of quenching is ~85 MPa and >350 MPa, respectively, which is a fourfold (~270 MPa) increase in strength, thereby making LA113 one of the highest specific-strength alloys reported due to its ultra-low density (1.4 g cm^−3^). Indeed, the yield stress of LA113 is ~330 MPa within 10 min of quenching. Such high strength observed so soon after quenching is supported by in situ Gleeble experiments involving solution treatment, water spray quenching and immediate compression (Supplementary Fig. 1). Both testing procedures infer that LA113 indeed shows immediate and substantial strengthening to a level not evident in any other age hardenable alloy system. It is demonstrated herein that such a phenomenon is caused by the rapid formation of a uniform distribution of rod-like, nano-sized and semi-coherent D0_3_-Mg_3_Al precipitates not reported previously.

### Microstructural characterization

**Fig. 2:**
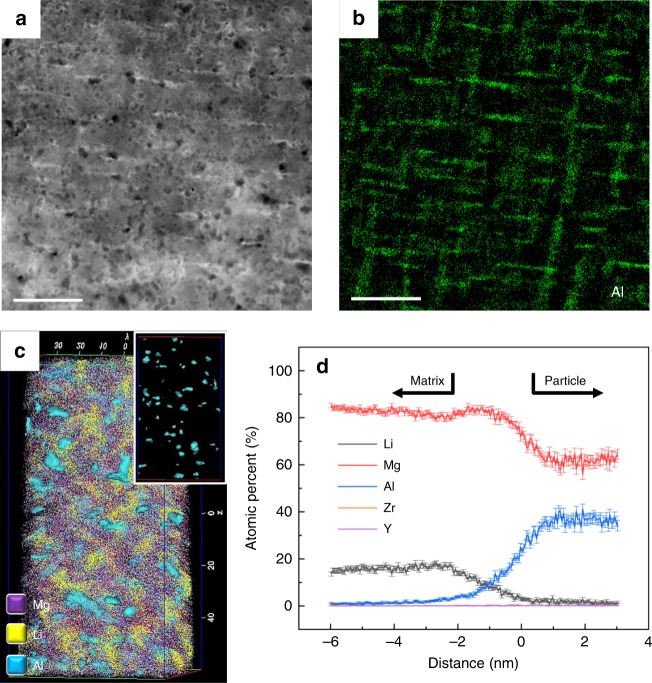
HIgh-angle annular dark-field (HAADF) scanning transmission electron microscope (STEM) images and atom probe tomography (APT) results of water-quenched (WQ) LA113. **a** STEM image with the corresponding Al compositional map shown in **b**. The rod-like precipitates are rich in Al. The fine particles with dark contrast are Li products induced by oxidation during sample transfer. **c** APT reconstruction of 70 × 70 × 120 nm^3^ volume, showing the distribution of Mg, Li and Al. The inset is the Al-rich precipitates highlighted by an iso-surface at a concentration of 15 at.% Al. **d** Proximity histogram showing the compositional changes through the Al-rich precipitates. The Mg to Al ratio of the precipitates is ~2, with only a small amount of dissolved Li. The error bars denote the standard deviation. Zr and Y are barely detectable from the APT data. Scale bar, 200 nm in **a**, **b**

Figure 1c, d shows typical bright-field transmission electron microscope (TEM) images of WQ L11 and LA113, respectively. The selected area electron diffraction (SAED) patterns of both alloys reveal a BCC β-matrix and diffraction spots corresponding to HCP α-Mg. Roughly spherical Mg particles of diameter ~50 nm are the only precipitate present in L11 (Fig. 1c). α-Mg is the usual phase to form in BCC Mg-Li alloys, where precipitates share a Burgers orientation relationship (OR) with the matrix: [$0\bar 11$]_β_ // [0001]_α_, ($\bar 211$)_β_ // ($\bar 1100$)_α_ (Supplementary Fig. 2). Conversely, the β-matrix of LA113 contains a uniform distribution of rod-like precipitates of ~10 nm in width and up to ~30 nm in length (Fig. 1d, e). The superlattice diffraction spots corresponding to these nano-sized precipitates are the {111} and {200} planes of a BCC-based D0_3_ ordered phase (the indexed and simulated SAED patterns are given in Supplementary Fig. 2 and 3). Hence, these D0_3_ precipitates share the “cube-on-cube” OR with the BCC matrix: <100>_β_ // <100>_D03_ and {110}_β_ // {110}_D03_. The dark-field TEM image in Fig. 1e, taken from the superlattice diffraction spot bounded by the red circle in the inset of Fig. 1d, confirms the rod-like morphology of this phase. The high-angle annular dark-field (HAADF) scanning transmission electron microscope (STEM) image of WQ LA113 (Fig. 2a) and the corresponding energy-dispersive spectroscopy (EDS) maps for Al (Fig. 2b) shows that these precipitates are also enriched in aluminium.

Atom probe tomography (APT) was used to quantitatively analyse the elemental distribution in LA113 soon after water quenching. The tomographic reconstruction in Fig. 2c reveals a very fine and uniform distribution of Al-containing zones (highlighted by an iso-concentration surface of 15 at.% Al). The proximity histogram in Fig. 2d displays the average compositional changes from the matrix to the Al-containing particles, revealing they are depleted in Li and have a Mg/Al ratio of around 2. A random selection of six precipitates within the 15 at.% iso-concentration surface (Table S1) reveals they are chemically similar, with an average composition (at.%) of 66.06 ± 3.97 Mg, 30.02 ± 5.15 Al and 3.92 ± 1.50 Li. Our combined TEM and APT analyses demonstrate that the rod-like precipitates in LA113 correspond to the D0_3_-Mg_3_Al ordered phase, containing a very limited amount of Li. The Mg/Al ratio from the APT analysis deviates from the exact stoichiometric composition (75 at.% Mg with 25 at.% Al), which suggests that some Al and Li atoms substitute for Mg atoms in the crystal structure of this phase.

**Fig. 3:**
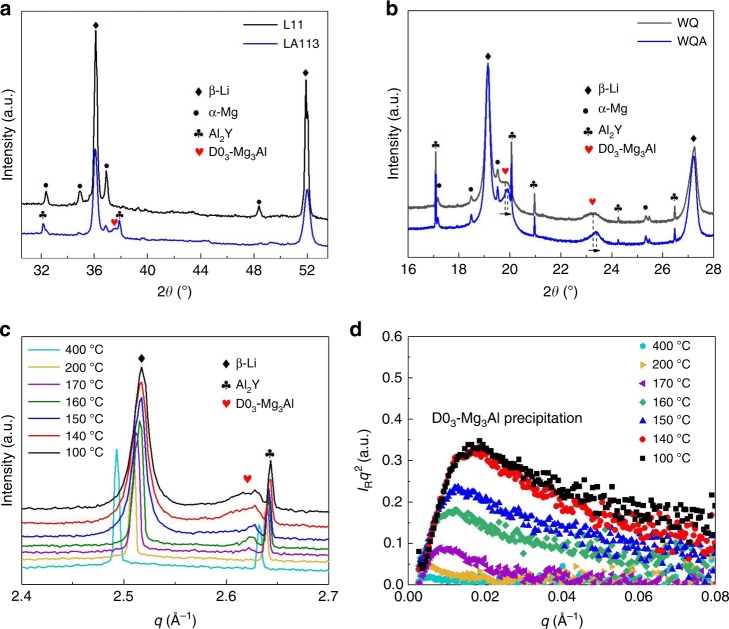
X-ray diffraction (XRD) profiles of L11 and LA113, and in situ X-ray synchrotron data of solution-treated LA113 during air quenching. **a** Cu Kα radiated XRD profiles of water-quenched (WQ) L11 and LA113, respectively. **b** Synchrotron XRD profiles of LA113 for the WQ and water-quench-aged (WQA) state, respectively. The 2θ angle of D0_3_-Mg_3_Al increases on ageing, indicating a decrease in *d*-spacings. **c** Wide-angle X-ray scattering (WAXS) profiles as a function of measuring temperature during air quenching, where *q* = 4*π*sin*θ*/*λ*, *θ* is half the scattering angle between the incident beam and the scattered beam and *λ* is the wavelength of the incident X-ray. The scattering peak corresponding to D0_3_-Mg_3_Al becomes visible at 170 °C with its intensity increasing during cooling to 140 °C. The peak shifts on cooling is induced by lattice thermal contraction. **d** Selected Kratky plots (*I*_*R*_*q*^2^ vs *q*) of the small-angle X-ray scattering (SAXS) data as a function of temperature on cooling, where *I*_*R*_ = (*I*_*T*_ − I_0_)/*I*_0_ is the relative change of the SAXS scattering intensity, *I*_*T*_ the scattering intensity at a given temperature *T* and *I*_0_ is the initial scattering intensity at 400 °C. The scattering from the D0_3_-Mg_3_Al phase first appears at 170 °C and increases to 140 °C, which matches closely with the WAXS results in **c**

The XRD profiles in Fig. 3a confirm that both WQ L11 and LA113 are indeed BCC (β-Li) alloys containing a very small volume fraction of HCP α-Mg phase (labelled), with LA113 also containing a small fraction of Al_2_Y intermetallics due to the preferential reaction of Y with Al during casting^7^. The diffraction peaks corresponding to the D0_3_-Mg_3_Al phase in LA113 are labelled. A high-resolution synchrotron XRD profile of WQ LA113 given in Fig. 3b shows the diffraction peaks corresponding to D0_3_-Mg_3_Al. There is notable peak broadening indicating that this phase is fine and strain-coupled with the BCC β-matrix. The lattice parameters of the BCC matrix and D0_3_-Mg_3_Al phases, calculated from the X-ray synchrotron data, are 3.51744 ± 0.000659 Å and 6.79998 ± 0.005630 Å, respectively (Supplementary Fig. 4)^22^.

**Fig. 4:**
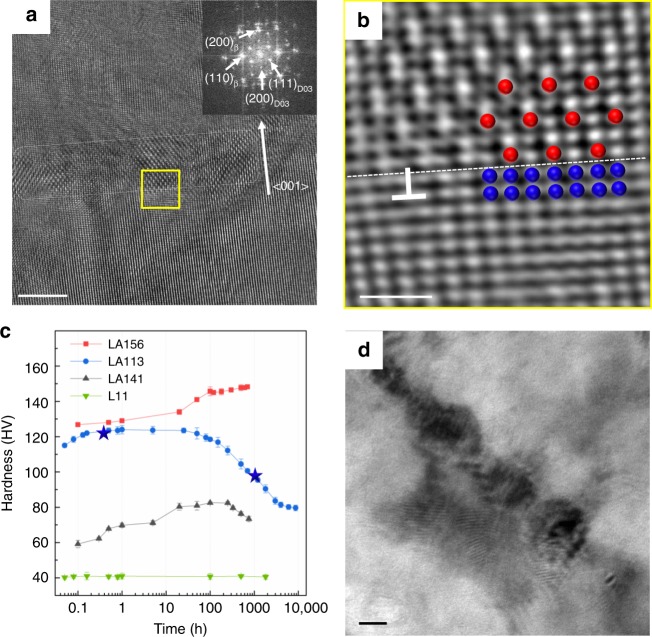
Transmission electron microscope (TEM) images of LA113, and the hardness of LA113 and L11 during natural ageing. **a** High-resolution TEM (HRTEM) images along [0 1 1]_β_ zone axis for water-quenched (WQ) LA113. The precipitate position is marked by the white dashed rectangle. The inset in **a** is the indexed fast Fourier transform (FFT). **b** The inverse fast Fourier transform (IFFT) pattern of the area marked by the yellow square in **a**. The white dashed line shows the interface between the precipitate and the matrix. The superlattice site and the matrix sites are inferred in red and blue dots, respectively. An interfacial dislocation is shown. **c** The hardness changes in WQ L11, LA113, LA141 and LA156 during natural ageing. Microstructures of LA113 natural aged for ~0.4 h and ~1000 h (marked by blue stars) are shown in **a**, **b** and **d**, respectively. The error bars denote the standard deviation. **d** Bright-field TEM image for WQA LA113. Scale bar, 5 nm in **a**, 1 nm in **b** and 10 nm in **d**

High-resolution TEM of WQ LA113, taken parallel to the <011>_β_ zone axis, was used for revealing the detailed structure of the D0_3_-Mg_3_Al phase (Fig. 4a, b). This rod-like phase is highlighted by the white dashed rectangle in Fig. 4a. The figure inset shows the fast Fourier transform (FFT) pattern, confirming its D0_3_ structure of this phase and the “cube-on-cube” OR with the matrix. The inverse fast Fourier transform (IFFT) pattern of the region marked by the yellow square in Fig. 4a is shown in Fig. 4b, revealing the periodic atomic columns of ordered D0_3_-Mg_3_Al (red dots) and the matrix (blue dots). The precipitate/matrix interface (white dashed line) is mediated by a mismatch dislocation (white). This interfacial dislocation is caused by the modest lattice mismatch between phases, calculated to be 3.3% using *δ* = (*a*_β_ − *a*_D03_/2)/*a*_β_, where *a*_β_ and *a*_D03_ are the lattice parameters of the matrix and D0_3_-Mg_3_Al, respectively. The value of *δ* and the observed interfacial dislocation indicates that the D0_3_-Mg_3_Al precipitates are semi-coherent with the BCC β-matrix. In general, lattice mismatch can create a certain degree of lattice distortion at the precipitate/ matrix interface, causing a classic coherency hardening effect. As D0_3_-Mg_3_Al is at least semi-coherent with the matrix, anti-phase boundaries are generated if the particles are sheared by gliding matrix dislocations. Thus, an order hardening effect is also expected to contribute to strengthening. Moreover, as the shear moduli of ordered phase are always higher than that of disordered matrix, there should also be a degree of modulus strengthening. The combination of these strengthening mechanisms in LA113 generates the substantial increase in the yield strength compared to L11.

However, while the strengthening induced by D0_3_-Mg_3_Al has now been addressed, the question arises: Does the D0_3_-Mg_3_Al phase form either during or very soon after quenching to generate the substantial, immediate strengthening, as seen in Fig. 1b (red curve)? This is investigated by time-resolved in situ synchrotron wide-angle/small-angle X-ray scattering (WAXS/SAXS) during rapid air cooling after solution treatment (Fig. 3c, d). Besides Al_2_Y, BCC β-matrix is the only phase present at 400 °C (Fig. 3c). During cooling, an X-ray peak corresponding to D0_3_-Mg_3_Al becomes evident at 170 °C that increases in intensity to 140 °C and then remains essentially constant. Figure 3d shows a Kratky plot (*I*_*R*_*q*^2^ vs. *q*) of the SAXS signal evolution as a function of temperature during cooling. Scattering becomes evident only when the temperature decreases to 170 °C, which correlates closely with Mg_3_Al formation seen using WAXS. The scattering peak positions also remain relatively constant, indicating that the volume fraction of Mg_3_Al rather than their size increases during cooling. The results demonstrate that D0_3_-Mg_3_Al forms at temperatures ranging from 170 °C to 140 °C and it is the only second phase observed during quenching. Compared to L11, the considerable increase in hardness/yield strength of LA113 soon after quenching makes it reasonable to conclude that this phase is responsible for the immediate hardening effect.

Although the atomic-scale structural changes in any alloy during an actual water quench are extremely difficult to capture, we must explain why D0_3_-Mg_3_Al forms at high temperature in LA113. There are several contributing factors. First, the solubility of Al in the BCC β-matrix decreases considerably as temperature decreases from 400 °C to 170 °C to below the Al content in LA113 (Supplementary Fig. 6), thereby generating a supersaturated solid solution on quenching and a high driving force to form Al-rich clusters^23^. Second, there is a very high rate of diffusion of Li atoms in a BCC Mg-Li matrix even at ambient temperature (extrapolated in Supplementary Fig. 7 to *D*_Li_ = 2.77 × 10^−9^ cm^2^ s^−1^ for Li in Mg-11Li^24^) that is associated with the expected high concentration of quenched-in vacancies (i.e., *C*_v_ ~10^−3^ in pure Li at 400 °C^25^). Finally, the actual strengthening phase identified in this work contains very little lithium (Supplementary Table 1), implying that the free energy of the system is minimized if Mg and Al aggregate rapidly under the assistance of the other two factors.

As demonstrated, Mg-Li-Al alloys such as LA113 significantly strengthen/harden immediately after quenching (Fig. 1). Figure 4c shows that LA113 hardens further for up to 0.2 h after quenching to a peak of ~120 Vickers Pyramid Number (HV) followed by a gradual decrease to ~95 HV after 1000 h (WQA: water-quenched and natural aged for 1000 h) that plateaus eventually at ~80 HV. The compressive stress–strain curve for natural aged LA113 (100 and 200 h) is presented in Supplementary Fig. 8. From the hardness and compression data for LA113, hardness and yield strength show an approximately linear relationship (i.e. *σ*_*y* = _2.83 HV). It is essential to understand the structural and chemical origin of this behaviour, as such alloys possess an unstable property profile. After 1000 h of natural ageing (WQA), the rod-like D0_3_-Mg_3_Al precipitates that form early in the hardening process show no change in crystal structure and minor compositional variations based on APT results (Supplementary Fig. 2 and 5). However, both coarsening and spheroidization of the initially rod-like precipitates is evident, as seen in Fig. 4d. The accumulation of interfacial stress and strain during coarsening gives rise to a loss of coherency and subsequent shape change to minimize surface energy. The X-ray synchrotron data reveal a decrease in *d*-spacings (Fig. 3b) and concomitant contraction of the D0_3_ unit cell, also implying a release of coherency strain. D0_3_-Mg_3_Al consequently becomes incoherent with the matrix and dislocations can only accumulate at the interface rather than shearing the particles. As a result, the coherency strain and order hardening effects are no longer applicable, leading to a reduction in strength/hardness. In contrast, L11 shows no discernible variation in its low hardness value at room temperature despite the observed precipitation of α-Mg, thereby indicating that age softening is not attributable to the formation of this phase.

From a thermodynamic viewpoint, to minimize the coherency strain energy of D0_3_-Mg_3_Al, it nucleates as rod-like particles that subsequently grow in a direction perpendicular to the elastically soft <001> directions (Fig. 4a). While this phase is also expected to be metastable based on phase equilibria considerations, we found no evidence of it transforming to a more stable phase even after 1000 h of natural ageing. It is pertinent to note that while AlLi (B32 ordered structure, space group: ${\mathrm{Fd}}\bar 3{\mathrm{m}}$, lattice parameter: 6.37 Å) is the Al-rich equilibrium phase in Mg-Li-Al^14^, its activation energy barrier for nucleation is much higher than D0_3_-Mg_3_Al because the latter is semi-coherent with the matrix (Fig. 4b), and hence has a relatively low interfacial energy. Indeed, the formation of this phase at high-energy incoherent interfaces is the result of the large difference in lattice parameter between AlLi and BCC matrix (~9.4%) and at temperatures above ~100 °C due to the considerable thermal activation required^13^.

## Discussion

While coarsening of the Mg_3_Al precipitates causes the alloy to soften with time, there are only minor compositional changes and a gradual loss of coherency with the β-matrix. Referring to the Lifshitz–Slyozov–Wagner (LSW) theory of Ostwald ripening, the three main factors affecting the rate of particle coarsening are particle/matrix interfacial energy and the solubility and diffusivity of matrix alloying elements, respectively^26,27^. Hence, it is necessary to minimize at least one of these factors for stabilizing the dispersion of nano-sized Mg_3_Al particles in these Mg-Li-Al alloys.

**Fig. 5:**
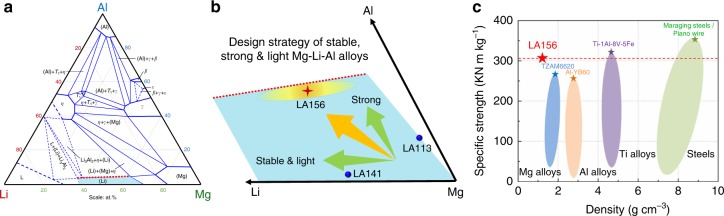
Design strategy for structurally stable, ultra-high-specific-strength body-centred cubic (BCC) Mg-Li-Al alloys. **a** Isothermal section of the Al-Li-Mg ternary system at 400 °C, adapted from SpringerMaterials. The single β-Li phase field is highlighted in light blue. **b** Schematic illustration of single β-Li phase area shown in **a**. The ternary alloy becomes more stable and less dense with increasing Li addition and stronger with more Al addition within the single β-Li region at 400 °C. The red dashed line shows the solubility limit of Al, whereby AlLi will form beyond it. The elliptic area provides a compositional guide for generating structurally stable, strong and light alloys. **c** Specific strength of LA156, compared with a large number of conventional Mg alloys, Al alloys, Ti alloys and steels. It is assumed that the yield strength of LA156 is *σ*_*y*_ = 2.83 Vickers Pyramid Number (HV)

The lattice parameter of the BCC β-matrix in Mg-Li binary alloys has been shown to decrease with increasing Li content^25,28^, thereby implying that the lattice parameter of the β-matrix of Mg-Li-Al alloys also decreases relative to that of the Mg_3_Al phase. The outcome is a decrease in the lattice mismatch between phases and a reduction in interfacial energy^19,29^. Hence, further addition of Li to LA113 will not only reduce density, but, according to the foregoing LSW theory, it will also create a more coarsening resistant particle distribution. Based on the evidence herein, nano-sized Mg_3_Al particles are the cause of the substantial strengthening in LA113 and, as such, an increase in Al content will increase the volume fraction of this phase, albeit to a certain degree since the maximum solubility of Al in BCC β at 400 °C (our solution temperature) is bounded by the red dashed line in Fig. 5a, b^30^. Aluminium contents greater than the maximum will simply form some incoherent AlLi particles that consume the excess Al and take Li out of the matrix. Hence, an increase in both Al and Li contents in our original LA113 (shaded elliptical region in Fig. 5b) will increase the volume fraction of nano-sized Mg_3_Al particles and reduce interfacial energy, thereby generating a higher strength alloy based on standard models of dispersion strengthening—the alloy also retains its strength over time since the particles are also resistant to coarsening. Based on these foregoing criteria, we designed a new Mg alloy containing 15 wt.% Li and 6 wt.% Al (designated as LA156, Fig. 5b). The lattice parameter of this alloy was calculated by laboratory XRD to be 3.48 Å, which is smaller than for LA113 under the same testing conditions (3.51 Å) and supports the argument that lattice parameter decreases with increasing Li content and a concomitant decrease in interfacial mismatch. This modified alloy strengthens immediately and naturally ages to a hardness of ~148 HV and last for over 700 h, which is 20 and 80% strength increase, and 4 times and 2 times more stable over LA113 and the commercial LA141 alloy, respectively (Fig. 4c). Taking the exceptionally low density of LA156 into consideration (~1.32 g cm^−3^), the new alloy is one of the highest specific-strength alloys ever developed (Fig. 5c)^31,32^.

In summary, the mechanisms of immediate precipitate strengthening on quenching and age softening due to the rapid formation and gradual coarsening of nano-sized, semi-coherent D0_3_-Mg_3_Al precipitates in Mg-11Li-3Al have now been explained. A new methodology for generating structurally stable, strong and light Mg-Li-Al alloys was proposed and validated. These findings provide new information for the design of second-generation ultra-lightweight, high-strength, corrosion-resistant Mg-Li-Al-based alloys with a more stable mechanical property profile compared with our recently discovered alloy^8^, thereby reducing the complexity of the processing route for manufacturing high specific-strength components suitable for use in various structural applications.

## Methods

### Specimen preparation

A magnesium-lithium based alloy with a composition of 30.30/10.95Li, 2.34/3.29Al, 0.039/0.19Zr and 0.128/0.59Y (at.%/wt.%), i.e. Mg-11Li-3Al-1(Zr, Y) (termed LA113 herein), was prepared by melting in an argon-protected electrical resistance furnace followed by casting. The as-cast alloy was homogenized for 8 h at 350 °C. Part of the homogenized alloy was extruded to plates of thickness 6 mm and width 60 mm. Rectangular samples with a size of 20 mm (ED) × 15 mm (TD) × 6 mm (ND) (ED extrusion direction, TD transverse direction, ND normal direction) were cut from the extruded plates for analyses of mechanical behaviour and microstructural development. Mg-11wt.% Li binary alloy (termed L11 herein), Mg-14wt.% Li-1wt.%Al ternary alloy (termed LA141 herein), Mg-15 wt.% Li-6 wt.%Al ternary alloy (termed LA156 herein) were melted in an induction furnace with an argon-protected atmosphere and cast into a copper mould. The as-cast alloys were homogenized for 4 h at 350 °C. All samples were heat treated in a tube furnace with argon protection for 10 min at 400 °C, followed by cold water quenching.

### Mechanical testing

Cylindrical samples of diameter 10 mm and height 20 mm (*l*/*d* = 2) were prepared from as-cast ingot for both L11 and LA113. Ambient temperature uniaxial compression testing was carried out using an Instron 5982 universal testing system operating at a true strain rate of 1 × 10^−4^ s^−1^. Vickers hardness testing was carried out along the central part of each sample, using a load of 1 kg and loading time of 15 s. At least 10 hardness indentations were generated for each sample. To investigate the strength generated in L11 and LA113 almost immediately after solution treatment and water quenching, in situ mechanical testing of 20 × 15 × 6 mm rectangular samples was conducted on a Gleeble 3500 Thermal and Mechanical Simulator. A type-K thermocouple was spot welded at the centre of the longitudinal plane of each sample. After heating and holding for 10 min at 400 °C, each sample was water spray quenched at ~ 200 °C s^−1^ followed by plane strain compression within 5 s of quenching to room temperature at a true strain rate of 2 × 10^−2^ s^−1^.

### Transmission electron microscopy

Heat-treated samples of L11 and LA113 were analysed by TEM. Thin foils of thickness 80 µm were prepared by mechanical polishing to 4000# SiC paper followed by ion-milling at −170 °C on a Gatan PIPS II Model 695 with vacuum of 2 × 10^−11^ Torr, which was used for avoiding natural ageing and oxidation. To capture the microstructure in the water-quenched state, thin foils are prepared within 10 min after water quenching and quickly transferred into PIPS at −170 °C as natural ageing is not expected at this low temperature. To minimize surface oxidation, TEM experiments were conducted within 5 min of sample preparation. TEM was carried out using three instruments, depending on the crystallographic features of the microstructures to be investigated. A Phillips CM200 TEM, operating at 200 kV, in conjunction with SAED, was used to investigate the general microstructure and orientation relationships between phases. A FEI TALOS FS200X G2 FEG TEM, operating at 200 kV, was used for HAADF scanning transmission electron microscope (STEM) imaging and EDS mapping. The angular range for HAADF detector is 46–200 mrad. High-resolution TEM was carried on FEI Themis-Z Double-corrected 60–300 kV S/TEM operating at 300 kV.

### Laboratory and synchrotron X-ray diffraction

Laboratory XRD experiments were carried out on a Panalytical MPD system using Ni-filtered Cu Kα radiation to investigate the phases present in the alloys. A 6 mm-thick sample was cut from the alloy and ground and polished to its 1/2 thickness. Each sample was adhered to a stainless steel sample holder and aligned with the X-ray beam. The voltage and current were 45 kV and 40 mA, respectively. The 2 theta scan range was 30 to 70°, scanning resolution was 0.05° per step and scanning speed was 1° per min. High-resolution synchrotron radiation experiments were carried out at the Powder Diffraction Beamline at the Australian Synchrotron using an X-ray wavelength of 0.826405 Å. All tests were conducted within 1 h after water quenching. The lattice parameters of the matrix and the precipitate phases in the water-quenched LA113 alloy were evaluated from synchrotron peaks by extrapolation method^22^. Time-resolved in situ SAXS/WAXS experiments were carried out at the SAXS/WAXS Beamline at the Australian Synchrotron using an X-ray wavelength of 1.52128 Å with the X-ray energy of 8.15 KeV.

### Atom probe tomography

APT was performed on a LEAP 3000X Si under green laser pulsing at a laser energy of 400 pJ and a repetition rate of 200 kHz for WQ as well as WQ and 1000 h natural aged (WQA) samples of LA113. The APT testing on the WQ sample was conducted within 6 h of quenching. APT samples were prepared from blanks of dimensions 0.5 × 0.5 × 15 mm by a two-step electropolishing procedure. The first step was carried out in an electrolyte of 25 vol.% perchloric acid in 75 vol.% acetic acid at 20 V and the second step was carried out in an electrolyte of 2% perchloric acid in 2-butoxyethanol at 20 V. During electropolishing, Li-rich zones were artefacts due to the acids used. Hence, Li-rich zones are not discussed within the main text. APT data were reconstructed using IVAS 3.6.8 software. The concentration of elements in the solute-rich zones of LA113 were calculated based on these identified solute-rich features. Proximity histogram analysis was utilized to investigate chemical composition across the iso-surface (15 at.% Al) of these solute-rich zones.

## Supplementary information

Supplementary Information

## Data Availability

The data that support the findings of this study are available from the corresponding authors upon reasonable request.
