# Supplementary material for: Precipitation strengthening in an ultralight magnesium alloy
Source: Nat Commun. 2019 Mar 1;10:1003. doi: 10.1038/s41467-019-08954-z (PMC6397269; doi:10.1038/s41467-019-08954-z)
Supplement: Supplementary file 1 — Supplementary Information [file 41467_2019_8954_MOESM1_ESM.pdf]

Supplementary Information

Precipitation strengthening in an ultralight magnesium alloy

Tang et al.

Supplementary Figures

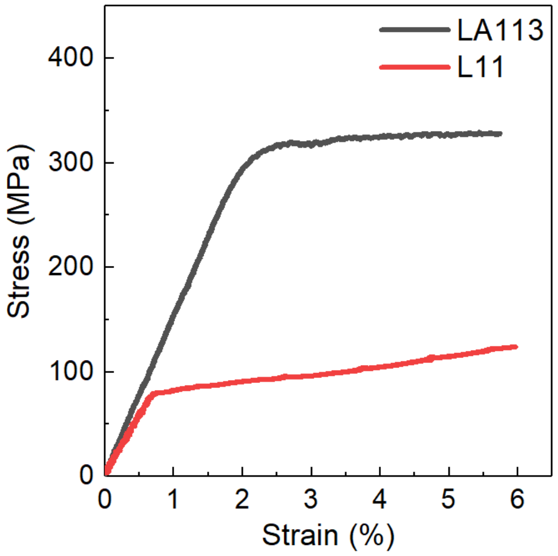

Supplementary Figure 1 | In situ Gleeble testing for L11 and LA113. Ambient temperature plane strain compression flow curves for both L11 and LA113, as generated in a Gleeble 3500 thermal and mechanical simulator. Test pieces were solution treated for 10 min at 400 ºC, water spray quenched and deformed within 5 s of quenching.

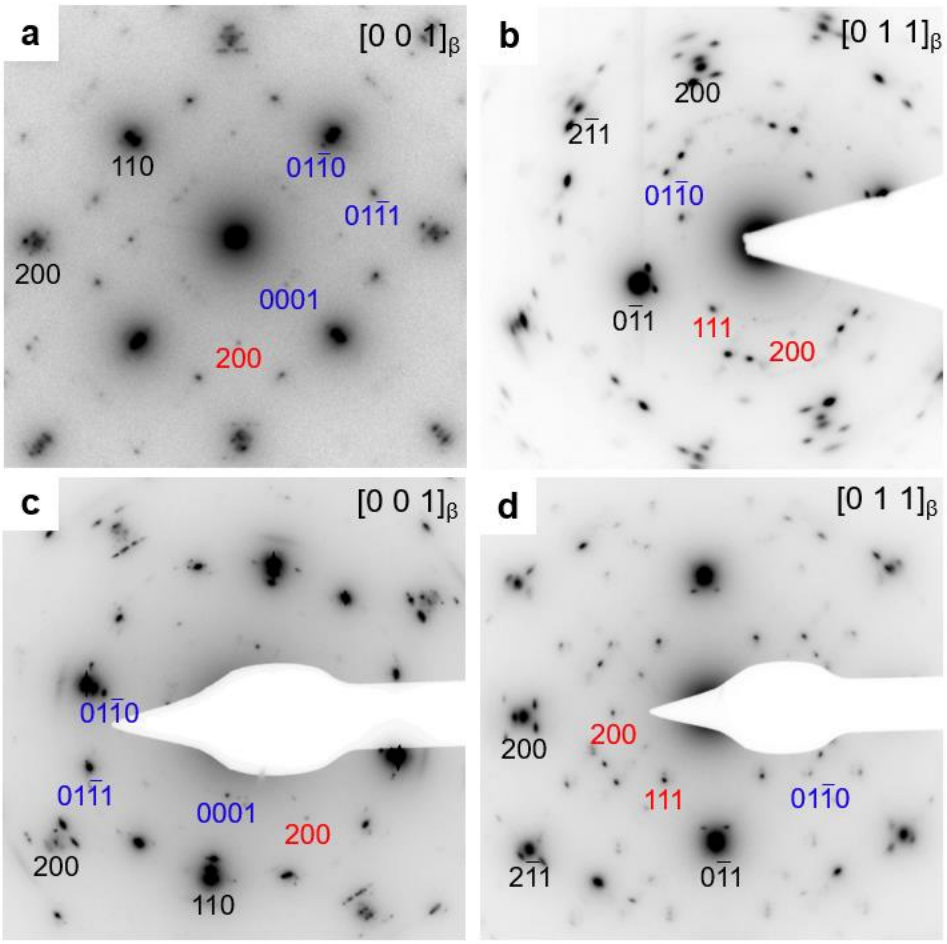

Supplementary Figure 2 | SAED patterns for WQ and WQA LA113. (a, b) Taken along $[0\ 0\ 1]_\beta$ and $[0\ 1\ 1]_\beta$ zone axes for WQ LA113. The indexing for BCC-matrix, HCP-Mg and $D0_3$-Mg_3Al are in black, blue and red, respectively. (c, d) Taken pattern along $[0\ 0\ 1]_\beta$ and $[0\ 1\ 1]_\beta$ zone axes for WQA LA113. Unindexed spots are induced by double reflection.

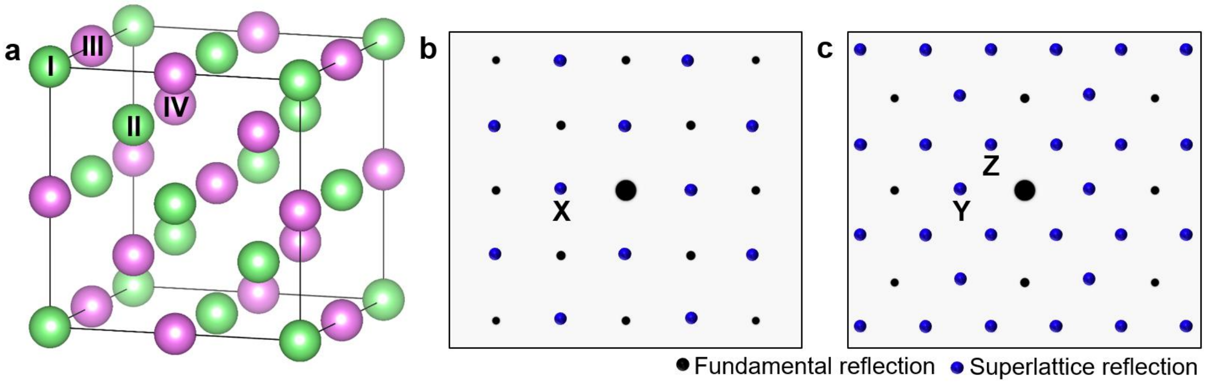

Supplementary Figure 3 | Three dimensional BCC-based ordered structures and simulated SAED patterns. (a) For B2-AB, sites I and III are A atoms, II and IV are B atoms; For B32-AB, sites I and II are A atoms, III and IV are B atoms; For $D0_3$-A_3B, site I is B atom, sites II, III and IV are A atoms. For $L2_1$-ABC_2, sites I and II are C atoms, site III is A atom, site IV is B atom. (b, c) Simulated SAED patterns of $[001]_\beta$ and $[011]_\beta$ zone axes, respectively. X, Y and Z represent the superlattice diffraction locations of {200}, {200} and {111} planes, respectively. For B2-AB, superlattice reflections are visible in locations X and Y; For B32-AB, superlattice reflections are only visible in location Z; For $D0_3$-A_3B and $L2_1$-ABC_2, superlattice reflections are visible in locations X, Y and Z;

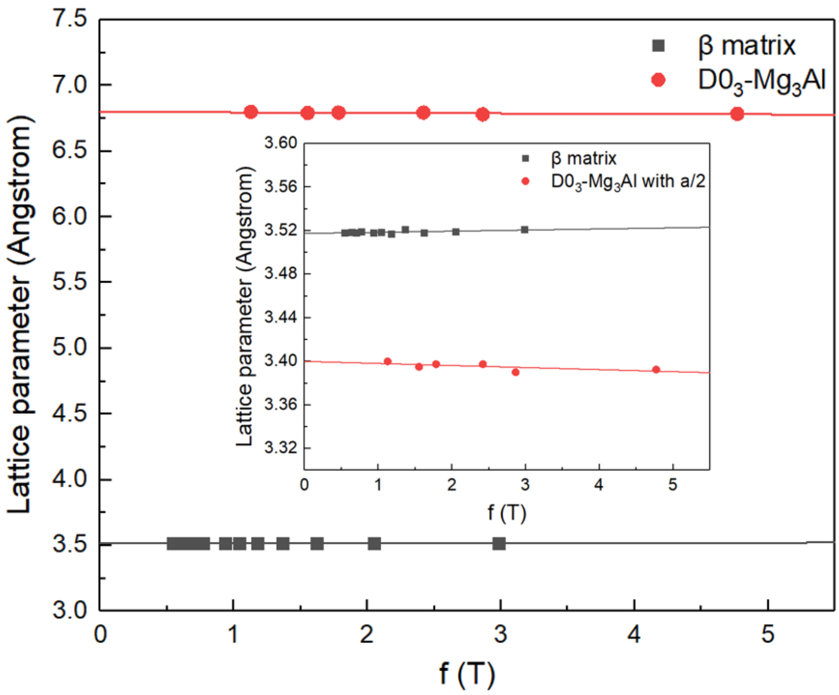

Supplementary Figure 4 | Lattice parameter of both the BCC β-matrix and D0$_3$-Mg$_3$Al phase as a function of f(T) in WQ LA113 alloy. $f(T) = [\cos^2(T)/\sin(T) + \cos^2(T)/T]/2$, where T is the Bragg angle for every peak. To minimize systematic error, the most reliable lattice parameters were obtained at f(T)=0 based on this extrapolation method. The inset shows the difference between the matrix lattice parameter and half the lattice parameter of D0$_3$-Mg$_3$Al phase, in order to calculate the lattice mismatch.

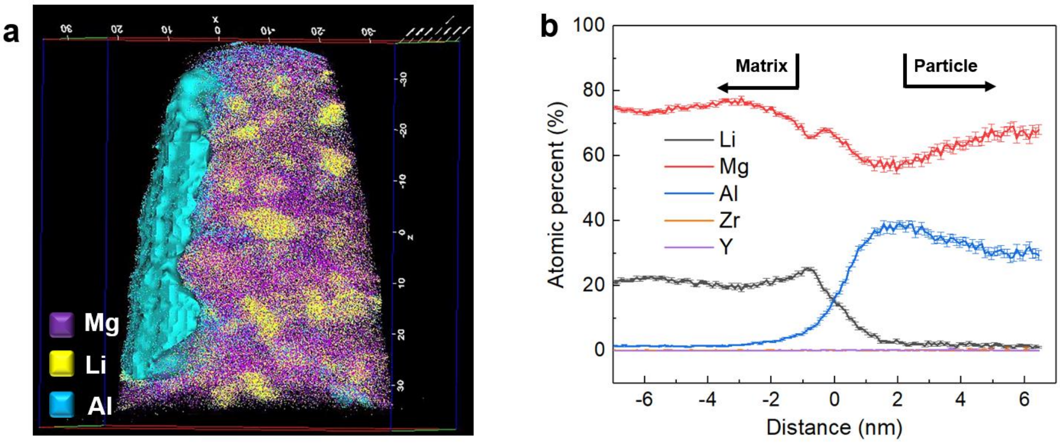

Supplementary Figure 5 | APT results of WQA LA113 alloy. (a) APT reconstruction of 70 x 70 x 70 nm^3 volume, showing the distribution of Mg, Li and Al. (b) Proximity histogram showing the compositional changes through the Al-rich precipitates. The error bars denote the standard deviation. Zr and Y are barely detected from the APT data.

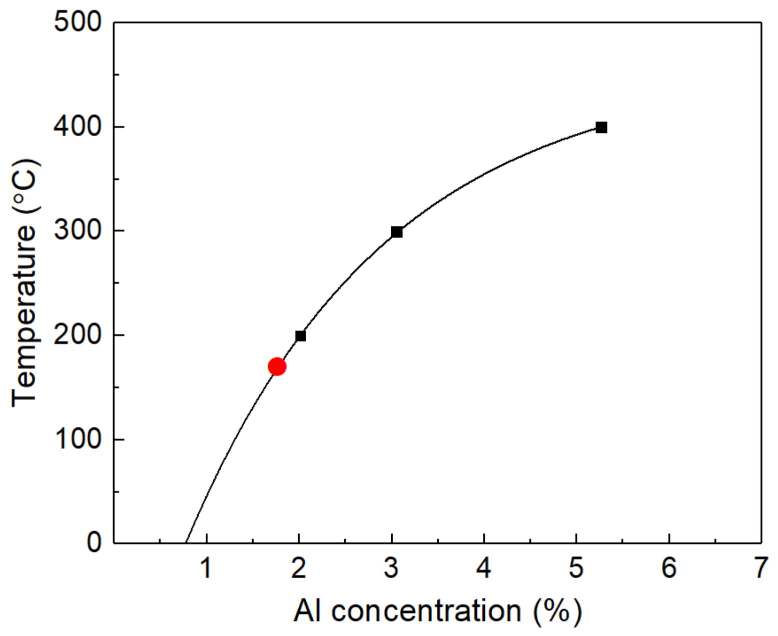

Supplementary Figure 6 | Solubility of Al in L11 at a function of temperature. The exponential fit is based on data taken from isothermal sections of the Mg-Li-Al ternary phase diagram, acquired from Springer Materials. The red dot show the extrapolated Al solubility (~1.8 at.%) at 170 ºC.

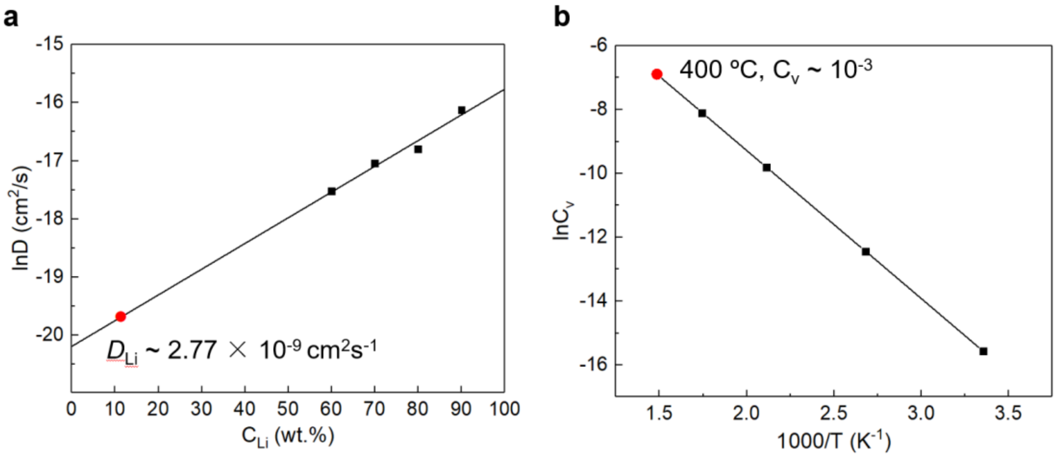

Supplementary Figure 7 | Diffusion coefficient and vacancy concentration of Li. (a) Ambient temperature diffusion coefficient of Li in Mg-11wt.%Li (red dot) extrapolated from a linear fit of the data (assuming $D_{Li} = D_o\exp(-Q/RT)$). (b) Equilibrium vacancy concentration in pure Li at ambient temperature (red dot) extrapolated from a linear fit of the data (assuming $C_V = C_o\exp(-Q/RT)$.

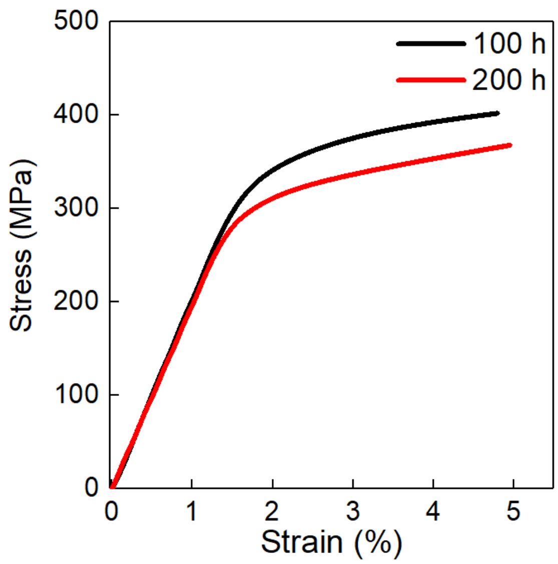

Supplementary Figure 8 | Compressive stress-strain curves for natural aged LA113. Ambient temperature stress-strain curves of LA113 tested in uniaxial compression after natural ageing for 100 h and 200 h, respectively.

Supplementary Table

Supplementary Table 1 | Chemical compositions of the Al-rich precipitates in WQ LA113.

Particle No.	Mg (at.%)	Al (at.%)	Li (at.%)
1	68.19	26.22	5.59
2	70.82	23.55	5.63
3	59.15	37.94	2.91
4	65.78	32.01	2.21
5	64.83	32.41	2.76
6	67.59	28.01	4.40
Average	66.06 ± 3.97*	30.02 ± 5.15	3.92 ± 1.50
* The errors denote one standard deviation from the mean.			
